# The COVID-19 pandemic - from great challenge to unique opportunity: Perspective^☆^^☆^

**DOI:** 10.1016/j.amsu.2020.08.037

**Published:** 2020-08-28

**Authors:** Irit Duek, Dan M. Fliss

**Affiliations:** Department of Otolaryngology Head and Neck Surgery and Maxillofacial Surgery, Tel Aviv Sourasky Medical Center, Sackler School of Medicine, Tel-Aviv University, Tel Aviv, Israel

**Keywords:** COVID-19, SARS-CoV-2, Infectious disease, Shared decision making, CoV, Coronavirus, SARS, severe acute respiratory syndrome, SDM, Shared decision making, WHO, World Health Organization

## Abstract

The 2019 novel coronavirus (SARS-CoV-2) and the disease it causes - coronavirus disease 2019 (COVID-19) have rapidly swept across the world since the first known human manifestation on December 8, 2019 in Wuhan (Hubei Province, China)1,2. The epidemic of the COVID-19 has presented as a grim and complex situation, causing great impact on economy and society, and seriously interfering with ordinary medical practice, threatening to exceed healthcare capacity in many countries over the globe. With no doubt, dealing with the COVID-19 has caused great social and medical crisis that presented great challenges to the medical and healthcare society, forcing it to face unprecedented times, and to reconceptualize how to provide quality health care while enforcing public health measures necessary for pandemic containment and optimal allocation of healthcare resources. However, along with this unparalleled time challenges, came great opportunities for changes and improvements, for innovations and creative solutions, some of which should be adopted and incorporated to the daily medical practices and social routine, even in the post-COVID-19 pandemic era.

## Highlights

• The 2019 novel coronavirus (SARS-CoV-2) has rapidly swept across the world.
• The epidemic of the COVID-19 has presented as a grim and complex situation, causing great impact on economy and society, and seriously interfering with ordinary medical practice.
• COVID-19 pandemic has created many challenges, but also forced us to re-examine how to provide more patient-centered high-quality care.
• The pandemic has changed how we deliver care, which also allows for re-evaluation and improvment of common practices.
• We should adopt some of the crisis' guidelines and habits to our new routine.

## Introduction

The 2019 novel coronavirus (SARS-CoV-2) and the disease it causes - coronavirus disease 2019 (COVID-19) have rapidly swept across the world since the first known human manifestation on December 8, 2019 in Wuhan (Hubei Province, China) [1,2]. The epidemic of the COVID-19 has presented as a grim and complex situation, causing great impact on economy and society, and seriously interfering with ordinary medical practice, threatening to exceed healthcare capacity in many countries over the globe. With no doubt, dealing with the COVID-19 has caused great social and medical crisis that presented great challenges to the medical and healthcare society, forcing it to face unprecedented times, and to reconceptualize how to provide quality health care while enforcing public health measures necessary for pandemic containment and optimal allocation of healthcare resources. However, along with this unparalleled time challenges, came great opportunities for changes and improvements, for innovations and creative solutions, some of which should be adopted and incorporated to the daily medical practices and social routine, even in the post-COVID-19 pandemic era.

### 1 Creative technological innovations and solutions

In the attempt to deal with the epidemic, which forced social distance and confronted us with equipment challenges (from protective measures to ventilator machines) creative innovations and solutions were developed. Masks and filters for ventilators were printed with 3D printers. To minimize contact with patients in the COVID-19 wards, remote monitoring of patients was implanted. Robotic equipment and technology were utilized to deliver medications and supplies to the hospitalized isolated patients. Bioinformatics and artificial intelligence is used, analysing “Big data”, developing mathematical algorithms for prediction of the spread of the epidemic, the natural history of the disease, infectivity, ways of transmission within populations, the number of ventilators that might be needed and the effectiveness of medications tried.

### 2 Telemedicine and remote collaborations

Internet access has become globally vital and essential during the last decades and its role cannot be emphasized enough during challenging times like these, when it is especially important to be able to remotely contact family, friends and to work from home. People lacking such access - particularly in developing countries - lack meaningful ways to influence and to be exposed to crucial relevant information.

### 2.1 Team meetings

In order to follow the social distance guidelines during the COVID-19 pandemic, clinicians and researches were encouraged to cease collocated meetings, and to alternatively utilize remote collaboration to continue group-based consultations, research projects and residents’ education.

Remote collaboration and medical staff meetings - professional and academic, were conducted virtually utilizing online and digital tools and technologies, such as web applications, which facilitates interdisciplinary medicine and research, while minimizing face-to-face contact with participants and still maintaining meaningful and productive working relationships and reciprocal co-learning. It has proven to be a legit feasible and effective alternative mode of communication, enabling easier meetings scheduling for many participants from distant locations, thus more comfortable and efficient while less time consuming. It also enables communicating easily with participants from distant departments and hospitals, creating interdisciplinary, trans-institutional, and cross-geographic collaboration, in order to advise, to share information and to learn, improving the management and treatment of patients who require thorough and professional discussion. Remote collaboration mechanisms may serve to be timely in facilitating, while also producing, interdisciplinary collaborative work. These resources can also be extended for future circumstances, post-COVID-19, as an alternative means of communication across distances, and when resources and capacity are limited.

### 2.2 Medical virtual visits - Ethics and equity

In effort to deal with the COVID-19 pandemic, the World Health Organization (WHO) provided fundamental mitigation and suppression strategies to reduce COVID-19 mortality and healthcare demand, which included social distancing, the postponement or cancellation of in-person gatherings [3] and working from home in isolation [4]. The pandemic has created both challenges and opportunities for delivering care, influencing the physician-patient relationship. While social distancing and health service reallocation can interfere with preference for an in-person visit, these measures also provide an avenue to study and implement virtual Shared decision making (SDM) processes, a management paradigm empowering patients as active partners in their own care, exchanging information and values, and optimizing the decision-making process [5]. Patients can be coached to assess the value of interventions, to trade-off benefits versus harms and assess their burdens, leading to new social norms in the clinical workplace. In person communicating risk at times of heightened uncertainty might pose a barrier to SDM engagement but also provides the opportunity to foster a patient-centered approach within a more personalized context. The epidemic advanced implementation of novel clinical engagement techniques such as digital tools to intervene remotely to reduce the negative effects of social isolation. While the encounter may be identical either in-person or virtually, many patients and clinicians are unfamiliar with the advantages of telehealth, or the risks of in-person care in a pandemic. Digital medicine gives us an opportunity to address the social determinants of health and provide access to everyone in their own neighbourhoods, so that not only the wealthy have the opportunity to be healthy. The possibility to advice with doctors all over the country and the world, enables patients from the periphery to advice with doctors from the centre, encouraging social egalitarianism. We must minimize face-to-face contact with vulnerable populations while persisting their treatment and health. Virtual meetings can in some cases replace office meetings and we are learning that it is possible to work more efficiently, less hours. It is unknown how significantly virtual visits impact the physician-patient relationship, nor its utility in those with low health literacy or those who are less technologically adept. Improvement in clinical outcomes with a virtual SDM telemedicine approach has been demonstrated [6]. A sub-analysis of a stratified randomized controlled trial showed that a telephonic SDM approach (incorporating telephonic coaching with decision aids that could be mailed, emailed or delivered online) resulted in lower medical cost, hospital admission rate, and fewer preference-sensitive interventions compared to the usual care group in patients with a variety of chronic health conditions such as diabetes, asthma, and chronic obstructive pulmonary disease [7]. The pandemic may foster changes that enhance care opportunities, team collaboration, and tools to reach underserved populations. Virtual remote medical visits have potential benefits that may modify healthcare and service allocation and reduce costs, by increasing the number of services/specialist access, reducing access disparities, reducing travel, lowering costs, and improving quality from more efficient information transfer. Such regulatory changes may enhance patient care and empowerment through more personalized, tailored, interactive encounters [8]. Careful and concerted efforts are needed to incorporate additional inputs and concerns that accompany virtual decisions, such as ethical issues expanded beyond the frequently considered constellation of privacy, security, confidentiality, liability. This unparalleled time presents some barriers to traditional medical visits given reduced face-to-face contact, but also an opportunity to evolve incorporation of SDM into the practices both during, and in the post-pandemic landscape. The context of a global pandemic provides a unique opportunity to re-examine how medical care is provided, and how patients are engaged as partners in their own medical decisions. Despite its many challenges, it is clear that this unprecedented period can provide opportunity to improve patients’ medical care.

### 2.3 Solidarity and collaborations

The pandemic created many blessed collaborations between scientific communities, medical centres and High-tech firms around the world, shearing information and knowledge. Rare collaborations were also conducted between different medical centres - national and international, yielding creative innovations and developments along with fruitful consultations, which improve and advance medicine towards the future. Creative partnerships and the digital economy can create a better world for all.

### 2.4 Research remote collaborations

Remote collaboration provides an opportunity for researchers to create and maintain international collaboratives and to create a collective knowledge base, providing an expansion of scientific inquiry and discovery [9,10]. The remote collaborations may lead to the establishment of personal and professional bonds among collaborating members and may provide valuable experience to lead future remote-based research projects, and to foster further interdisciplinary collaboration. Remote collaboration enables researchers in physical and geographic distances to act swiftly and collectively to address emerging and unexpected challenges, such as working together under public health restrictions that include distancing during a pandemic, while maximizing the value and utility of existing accessible and cost-effective communication technologies. Beyond COVID-19, remote collaboration may equip research teams and learning health systems with tools and capacities that can be used to leverage digital technology in producing innovative and collaborative work, while facilitating and sustaining meaningful effective and productive long-distance working relationships, when physical proximity is not possible. Remote collaboration plays a crucial role under conditions of scarcity and crisis in health systems. The innovation in the remote collaboration approach is not the technologies that facilitate it, but the ability to adapt and flex under changing, unpredictable and challenging conditions.

### 3 Less emergency department admissions

The acknowledgment that the hospital isn't the sole and central solution for all medical treatments. Many medical situations can be solved via telemedicine, by virtual visits. Hospital admissions should be reserved for complex treatments, requiring unique equipment and trained qualified crews. Many other situations can be solved from distance or in the community primary clinics. Implanting this recognition will be better for the patients, for the medical system and for the nation economy. Available tele-health has allowed patients to be screened and supported at home, giving the hospitals time to prepare for the onslaught of intensive care treatment for those who need it.

### 4 Increased efficiency of the medical system - smaller crews

The social distance guidelines forced us working in 12 h shifts, and in small crews including less personnel than usual. We have proved it is possible to shorten the residents’ shifts, work more efficiently, less hours.

### 5 Awareness to the need for hygiene and protection measures from aerosol

The medical protection measures required in the outpatient clinic as well as in operation ward in otolaryngology head and neck department, which aim to protect medical staff from COVID-19 infection, should be adopted for protection from aerosol of other daily infective agents.

### 6 Encouraging hygiene and vaccination, diminishing infective diseases

It is well known that low hygiene is the direct cause of all sort of infectious diseases outbreaks, as well as intestine infections, upper respiratory infections, Influenza and pneumonia, which cause great burden upon the medical system, economy, and increased mortality. The pandemic may increase the awareness for importance of hygiene as well as routine vaccinations and thus may increase vaccination compliance. Meticulous maintenance of personal and public hygiene along with increased vaccinations compliance could decrease morbidity and improve public health.

### 7 Quick adaptation and flexibility

This epidemic forced the medical system and community, which is sometimes based upon traditional habits and guidelines, not easily and not often changing, to be conducted with quick adaptation to new, unknown and changing reality. Also, facing a novel unknown virus causing unpredicted disease, forced changing the guidelines every few days, which required tremendous flexibility, that should be implanted in our daily routine for good.

### 8 Increasing the health care system budgets

This epidemic has found many health care systems around the world unprepared, unequipped and underbudgeted, lacking the ability to assemble a powerful intensive care capacity. The sudden great demand for medical equipment, protective measures, ventilators, intensive care units’ beds, has led some health some Western health systems to collide. Perhaps a lesson to be learned is the crucial need for adequately budjeted, well equipped health care systems during routine, in order to avoid collision in face of medical crisis, being prepared towards every possible challenging scenario.

### 9 Developing vaccination to corona virus

The pathogen SARS-CoV-2 is a positive-sense RNA, 29903-bp betacoronavirus, first isolated in the Wuhan seafood market on January 7, 2020 [11]. It is highly homologous to the previous severe acute respiratory syndrome (SARS) coronavirus (CoV) (SARS-CoV-1)^1^, that has emerged two decades ago. The funding for vaccination for the SARS-CoV-1 ended before vaccination was developed. It is possible that SARS-CoV-1 vaccination could have aid to shorten the period currently required to develop vaccination to the novel SARS-CoV-2. It is reasonable to assume that the vaccination, medications, scientific findings and other medical and technological solutions that will soon be developed to manage with the COVID 19, will aid dealing with the next future epidemic that might struck.

## Conclusions

COVID-19 pandemic has created many challenges, but also forced us to re-examine how to provide more patient-centered and high-quality care. The pandemic has changed how we deliver care, which also allows for re-evaluation of common practices and enhancement of management strategies effectiveness. The COVID-19 pandemic will eventually pass. It will leave behind grief, destruction and economic crisis. However, along with the sorrow and great challenges, we have a unique opportunity to make an impact on the medical society now, as this pandemic continues to unfold, and hopefully, in the day after. If we succeed to learn from this epidemic and from the way we deal with it, perhaps many things will change for the better. Instead of returning to our previous routine, we should adopt some of the crisis’ guidelines and habits to our new innovative, creative, solidary, compassionate, effective, clean, equal, healthy, better routine.

### Provenance and peer review

Not commissioned, editor reviewed.

## Annals of medicine and surgery

The following information is required for submission. Please note that failure to respond to these questions/statements will mean your submission will be returned. If you have nothing to declare in any of these categories then this should be stated.

## Ethical approval

N/A.

## Please state any sources of funding for your research

N/A.

No funding sources to declare.

## Author contribution

Irit Duek - study concept and design, data collection, data analysis and interpretation, writing the paper.

Dan M. Fliss - study concept and design, paper review.

## Please state any conflicts of interest

N/A.

The authors have no financial conflicts of interest relevant to this article to disclose.

## Registration of research studies

1. Name of the registry:
2. Unique Identifying number or registration ID:
3. Hyperlink to your specific registration (must be publicly accessible and will be checked):

## Guarantor

Irit Duek.

## Consent

N/A.

## Declaration of competing interest

The authors have no financial conflicts of interest relevant to this article to disclose.
